# Cycles of vascular plexus formation within the nephrogenic zone of the developing mouse kidney

**DOI:** 10.1038/s41598-017-03808-4

**Published:** 2017-06-12

**Authors:** David A. D. Munro, Peter Hohenstein, Jamie A. Davies

**Affiliations:** 1 Centre for Integrative Physiology, The University of Edinburgh, Hugh Robson Building, 15 George Square, Edinburgh, EH8 9XD United Kingdom; 2 The Roslin Institute, The University of Edinburgh, Easter Bush, Edinburgh, EH25 9RG United Kingdom

## Abstract

The renal vasculature is required for blood filtration, blood pressure regulation, and pH maintenance, as well as other specialised kidney functions. Yet, despite its importance, many aspects of its development are poorly understood. To provide a detailed spatiotemporal analysis of kidney vascularisation, we collected images of embryonic mouse kidneys at various developmental time-points. Here we describe the first stages of kidney vascularisation and demonstrate that polygonal networks of vessels (endothelial plexuses) form in cycles at the periphery of the kidney. We show that kidney vascularisation initiates at E11, when vessels connected to the embryonic circulation form a ring around the ureteric bud. From E13.5, endothelial plexuses organise around populations of cap mesenchymal and ureteric bud cells in a cyclical, predictable manner. Specifically, as the ureteric bud bifurcates, endothelia form across the bifurcation site as the cap mesenchyme splits. The plexuses are vascular, carry erythrocytes, are enclosed within a basement membrane, and can always be traced back to the renal artery. Our results are a major step towards understanding how the global architecture of the renal vasculature is achieved.

## Introduction

Adult mouse kidneys receive 9–22% of cardiac output^1–3^. Most of this blood travels into the glomerular capillaries in the renal cortex^4, 5^ where small molecules are filtered from the plasma. To filter blood, the kidneys must develop an extensive and intricately arranged vascular system during development, and the function and health of these vessels must be preserved throughout adulthood.

The development of the metanephric kidney begins at E10.5 when an epithelial tubule evaginates from the caudal portion of the Wolffian/nephric duct^6^. This epithelial outgrowth is the ureteric bud, a structure that develops into the ureter and collecting ducts of the adult kidney. The ureteric bud invades the metanephric mesenchyme (a mass of undifferentiated cells derived from the posterior intermediate mesoderm) at approximately E10.5-11, and starts to branch shortly thereafter. As it branches, progenitor cells within the metanephric mesenchyme are induced to differentiate. The nephrogenic progenitors, which are condensed around each ureteric bud tip (within the cap mesenchyme)^7, 8^, are prompted to undergo mesenchymal-to-epithelial transitions, resulting in the initiation of nephron formation. The inductive interactions between the ureteric bud and the cap mesenchyme are well understood^9^, but the mechanism by which endothelia organise alongside these cell types is less well characterised.

Blood vessels can form via vasculogenesis or angiogenesis. Vasculogenesis is the *de novo* formation of blood vessels via the differentiation and coalescence of endothelial precursors^10, 11^ and angiogenesis is the formation of new blood vessels via sprouting or splitting from pre-existing vessels^12, 13^. A major concept proposed to explain kidney vascularisation purports that the kidney is vascularised via a combination of vasculogenic and angiogenic processes^14–18^. According to this theory, blood vessels arise *in situ* via vasculogenesis around the periphery of the kidney from endogenous endothelial progenitors, whereas the endothelia of the major vessels and the medulla form via angiogenesis, by branching from extrinsic sources. Eventually, these angiogenic vessels are thought to communicate and connect with the vasculogenic vessels. Early support for this model came from Colberg (1863)^19^, who provided evidence for the existence of glomeruli not connected to the circulation, and Herring (1900)^20^, who proposed that blood vessels can develop endogenously in the kidney. More recently, this concept has been substantiated by studies that have identified endogenous endothelial and mural cell progenitors within the metanephric mesenchyme^21–25^: this implies that the kidney has the potential to form blood vessels intrinsically. But this model of kidney vascularisation remains unproven, ambiguous, and somewhat controversial.

To better understand the relationship between endothelial and epithelial organisation during kidney development, we analysed high-resolution images of normal CD-1 mouse kidneys that had developed *in vivo* from the initiation of metanephric organogenesis to birth. We demonstrate that the early embryonic kidney initially becomes vascularised via systemically connected blood vessels and that endothelia are patterned at the border of the kidney in a cyclical, non-stochastic manner. Collectively, these data provide a new conceptual model to explain how the renal blood vessels develop.

## Results

### The first renal blood vessels arise from a peri-Wolffian mesenchymal region and form a vascular ring around the ureteric bud stalk

Although previous studies have investigated the initial phases of kidney vascularisation^21, 25–28^, the precise spatiotemporal origin of the first renal blood vessels remains unknown. By taking confocal images of whole-mount kidneys at various developmental time points with anti-CD31 (endothelial marker) and anti-laminin (ureteric bud basement membrane marker), we have mapped the first steps of kidney vascularisation in the CD-1 mouse embryo.

**Figure 1:**
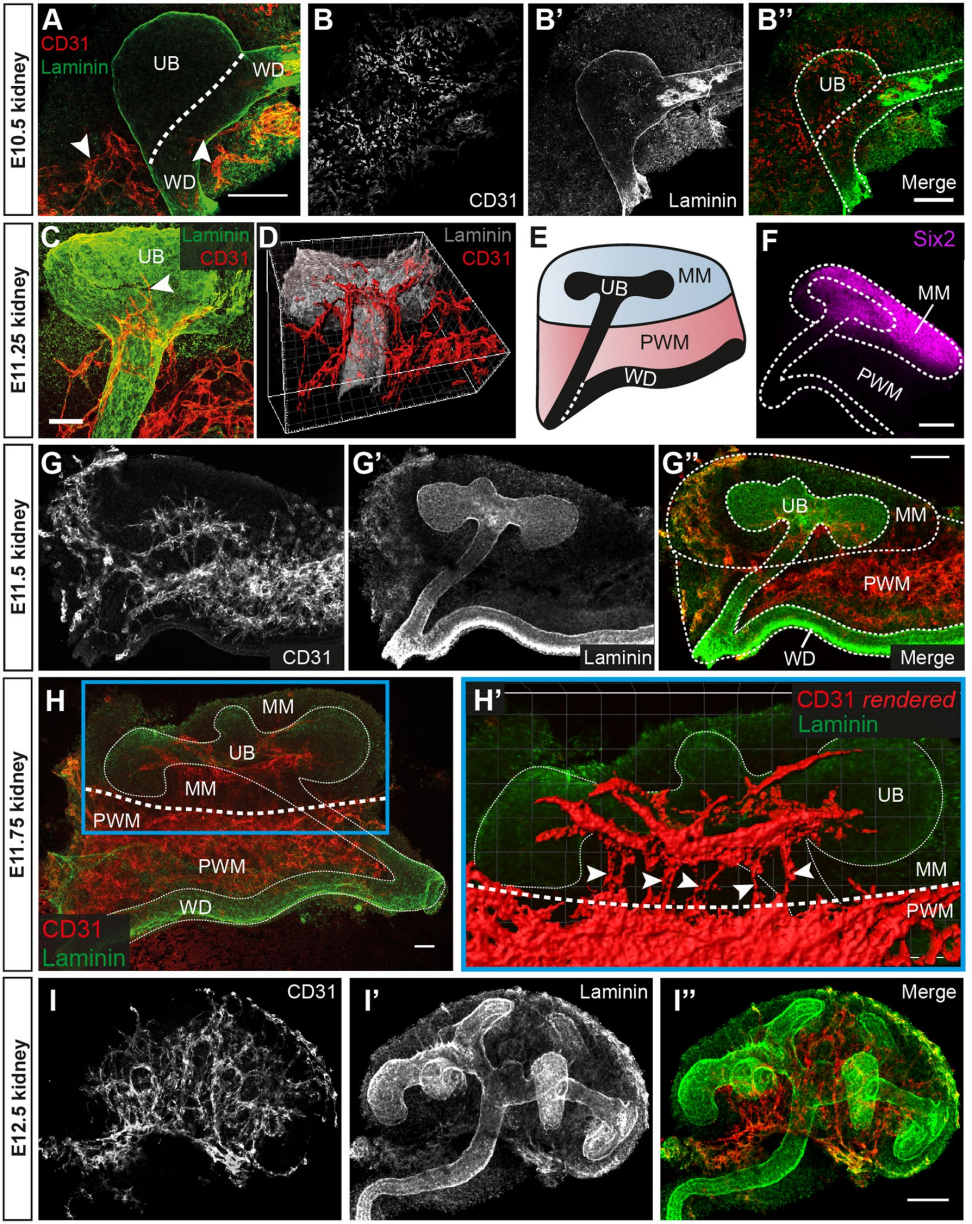
Development of the first renal blood vessels. (**A**) At E10.5, CD31 expressing capillaries (arrowheads) are present adjacent to the ureteric bud (UB) and (**B**-**B”’**) the metanephric mesenchyme is surrounded by scattered endothelia. (**C**,**D**) By E11.25, Blood vessels had formed a vascular ring around the top of the ureteric bud stalk, and cell projections extended into the metanephric mesenchyme (arrowhead; D is a 3-D rendered version of C). (**E**) Cartoon representing the different tissue regions discussed in this section. (**F**) Immunostaining for Six2 in the E11.5 metanephric mesenchyme and peri-Wolffian mesenchyme illustrates that these tissue regions are distinct. (**G**-**G”**) By E11.5, blood vessels had begun to invade the metanephric mesenchyme, always entering from a peri-Wolffian mesenchymal region. (**H**-**H’**) Blood vessels continue to invade the metanephric mesenchyme by E11.75 from the peri-Wolffian mesenchyme (H’ shows the region in the blue box in H; white arrowheads in H’ show connection between peri-Wolffian and metanephric mesenchymal blood vessels). (**I**-**I”**) By E12.5, the metanephric mesenchyme is vascularised throughout. UB, ureteric bud; WD, Wolffian duct; MM, metanephric mesenchyme; PWM, peri-Wolffian mesenchyme. Scale bars: (A–E) = 100 µm; (G–M) = 50 µm.

The ureteric bud begins to develop at E10.5 as it evaginates from the Wolffian duct and invades the metanephric mesenchyme, marking the initial phase of metanephric organogenesis. Confocal z-projections of the E10.5 kidney show CD31^+^ capillaries to be present adjacent to the ureteric bud (Fig. 1A). At this time, the core of the presumptive metanephric mesenchyme is avascular, but scattered CD31^+^ endothelia, which have not connected to form vessels, can be detected around its border (Fig. 1B-B”), in agreement with previous reports^26, 28^.

The ureteric bud continues to extend from the Wolffian duct between E10.5 and E11.25 before undergoing its first bifurcation (forming a T-bud; Fig. 1C). By E11.25, CD31^+^ endothelial vessels have formed along the length of the ureteric bud and have arranged as a vascular ring around the top of the stalk (Fig. 1C,D). The metanephric mesenchyme remains predominantly avascular at this time, but it is notable that endothelial cell projections extend from the vascular ring towards the body of the kidney (Fig. 1C).

By E11.5, endothelial vessels had started forming within the metanephric mesenchyme: these vessels always entered the kidney from a capillary dense region that lies between the Wolffian duct and the metanephric mesenchyme, which we refer to as the peri-Wolffian mesenchyme (Fig. 1E-G”).

Considering the E11.5 ureteric bud as a ‘T’ shape, the cross-stroke of the ‘T’ elongated before its next round of branching initiated. While this elongation took place, many blood vessels had formed within the metanephric mesenchyme (Supplementary Fig. 1). Consequently, by E11.75 the metanephric mesenchyme was largely vascularised, and 3D rendering showed that blood vessels were still connected to the blood vessels of the peri-Wolffian mesenchyme (in 8/8 kidneys analysed; Fig. 1H-H’). Moreover, blood vessels had developed in connection with the vascular ring surrounding the top of the ureteric bud stalk (Movie 1). By E12.5, blood vessels had formed throughout the metanephric mesenchyme (Fig. 1I-I”).

Collectively, these data demonstrate that the first renal blood vessels formed adjacent to the ureteric bud by E11.25, making a vascular ring around the top of the stalk. These vessels arose from the peri-Wolffian mesenchyme, and the entire metanephros was vascularised by E12.5.

### The first renal blood vessels carry erythroid cells and connect to the embryonic circulation via the caudal and common iliac arteries

In congruence with our work, a recent study has demonstrated that the E11.5 embryonic kidney contains endothelial vessels^26^ but, to our knowledge, no studies have determined whether these early endothelial vessels are isolated or if they connect with larger vessels that are outside of the kidney.

**Figure 2:**
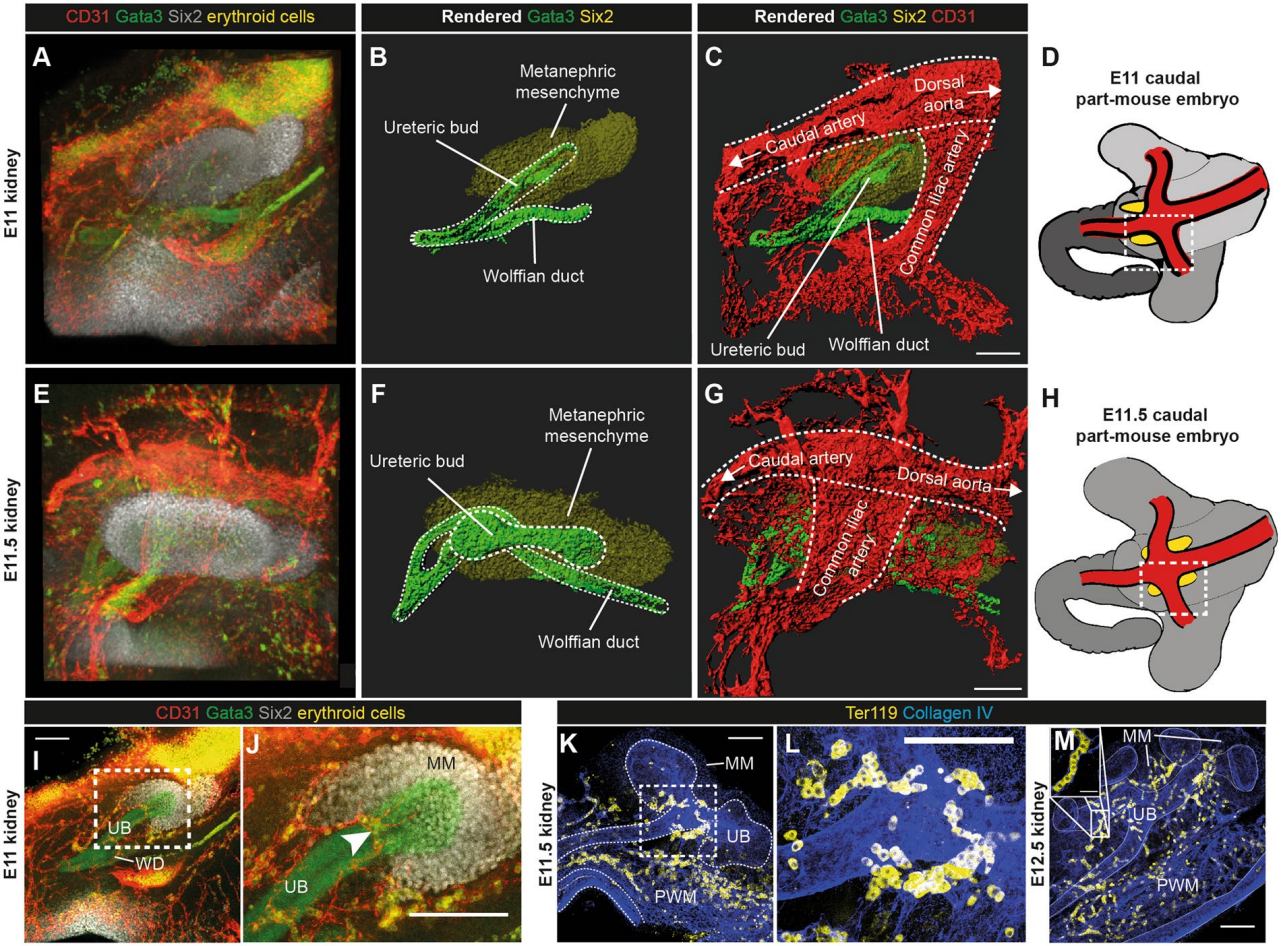
The E11 and E11.5 kidneys are vascularised by blood vessels that carry erythroid cells and connect to the caudal and common iliac artery. (**A–D**) E11 kidney and the surrounding tissue. (**B**) Rendered image from A, showing the E11 metanephric mesenchyme, ureteric bud, and Wolffian duct. (**C**) Rendered image from A, showing the location of the major blood vessels in relation to the kidney. (**D**) Cartoon illustrating the region displayed in A-C (dashed white box) in relation to the caudal part of the mouse embryo (kidneys in yellow). (**E-H**) E11.5 kidney and the surrounding tissue. (**F**) Rendered E11.5 kidney, from E. (**G**) Rendered kidney and major blood vessels, from E. (**H**) Cartoon illustrating the region displayed in E-G (dashed white box) in relation to the caudal part of the mouse embryo (kidneys in yellow). (**I–L**) Erythroid cell carrying blood vessels form a ring around the ureteric bud at E11 and E11.5. Arrowhead in J shows the vascular ring carrying erythroid cells. (**M**) The E12.5 kidney is vascularised by blood vessels carrying erythroid cells (inset image in M shows that the erythroid cells in the E12.5 kidney are nucleated). UB, ureteric bud; WD, Wolffian duct; MM, metanephric mesenchyme; PWM, peri-Wolffian mesenchyme. Scale bars: 100 µm.

To examine whether the first renal vessels are connected to the embryonic circulation, we immunostained the caudal regions of E11 and E11.5 mouse embryos for CD31 (endothelial marker), Gata3 (ureteric bud marker), and Six2 (cap mesenchyme marker) followed by tissue clearing with benzyl alcohol/benzyl benzoate (BABB) and confocal microscopy. At E11, the cranial pole of the kidney lies ventrally to the common iliac artery and the dorsal aspect of the kidney lies ventrolaterally to the caudal artery (Fig. 2A–D; Movie 2). The vascular ring had already formed around the ureteric bud stalk by E11 and it contained loose suspended cells that resembled erythroid cells (Fig. 2I,J; Movie 3). These loose suspended cells were also abundantly found within the major arteries. As BABB clearing does not act to remove the heme from haemoglobin, and because heme acts as a major chromophore under visible light^29, 30^, the observation of erythroid cells was unsurprising. These cells fluoresced under green and red light, so they appeared as yellow. We verified that these were erythroid cells by co-localisation analyses with the erythroid marker Ter119 (Supplementary Fig. 2). Additionally, conventional whole-mount staining for Ter119 at E11.5 and E12.5 verified that the early renal blood vessels carry red blood cells (Fig. 2K–M; Movie 4).

In the mouse embryo, primitive erythroblasts are gradually enucleated between E12.5 and E16.5, resulting in the generation of mature embryonic erythrocytes^31, 32^. In agreement with these studies, the Ter119^+^ cells within the E11.5 and E12.5 kidneys were nucleated (100% of Ter119^+^ cells were nucleated at E11.5 [*n* = 100 cells] and E12.5 [*n* = 100 cells]; for both ages, the 95% confidence intervals are ± 0.5%), indicating that they are primitive erythroblasts (Fig. 2K–M; Movie 4).

Scrolling through confocal z-planes of the caudal region of the E11 mouse demonstrated that the blood vessels of the vascular ring could be traced to either the caudal or common iliac arteries, so the vessels of the E11 kidneys must have been connected to the embryonic circulation (Movie 3).

By E11.5, the kidney had migrated caudally, and now lay directly ventrally to the common iliac artery (Fig. 2E–H; Movie 5). Although the metanephric mesenchyme was predominantly avascular at E11.5 (with the exception of the blood vessels around the ureteric bud), it was completely surrounded by blood vessels (Supplementary Fig. 3). Confocal z-planes of the caudal portion of the E11.5 mouse demonstrated that the blood vessels around the ureteric bud connected to either the caudal or common iliac arteries (Supplementary Fig. 3C) and that these blood vessels contained erythroid cells (Movie 6).

Together, these data demonstrate that the blood vessels of the E11 and E11.5 kidneys are connected to the embryonic circulation via the caudal and common iliac arteries and that these blood vessels carry primitive erythroblasts.

### Endothelial plexuses form around ureteric bud tips in the nephrogenic zone of the kidney

**Figure 3:**
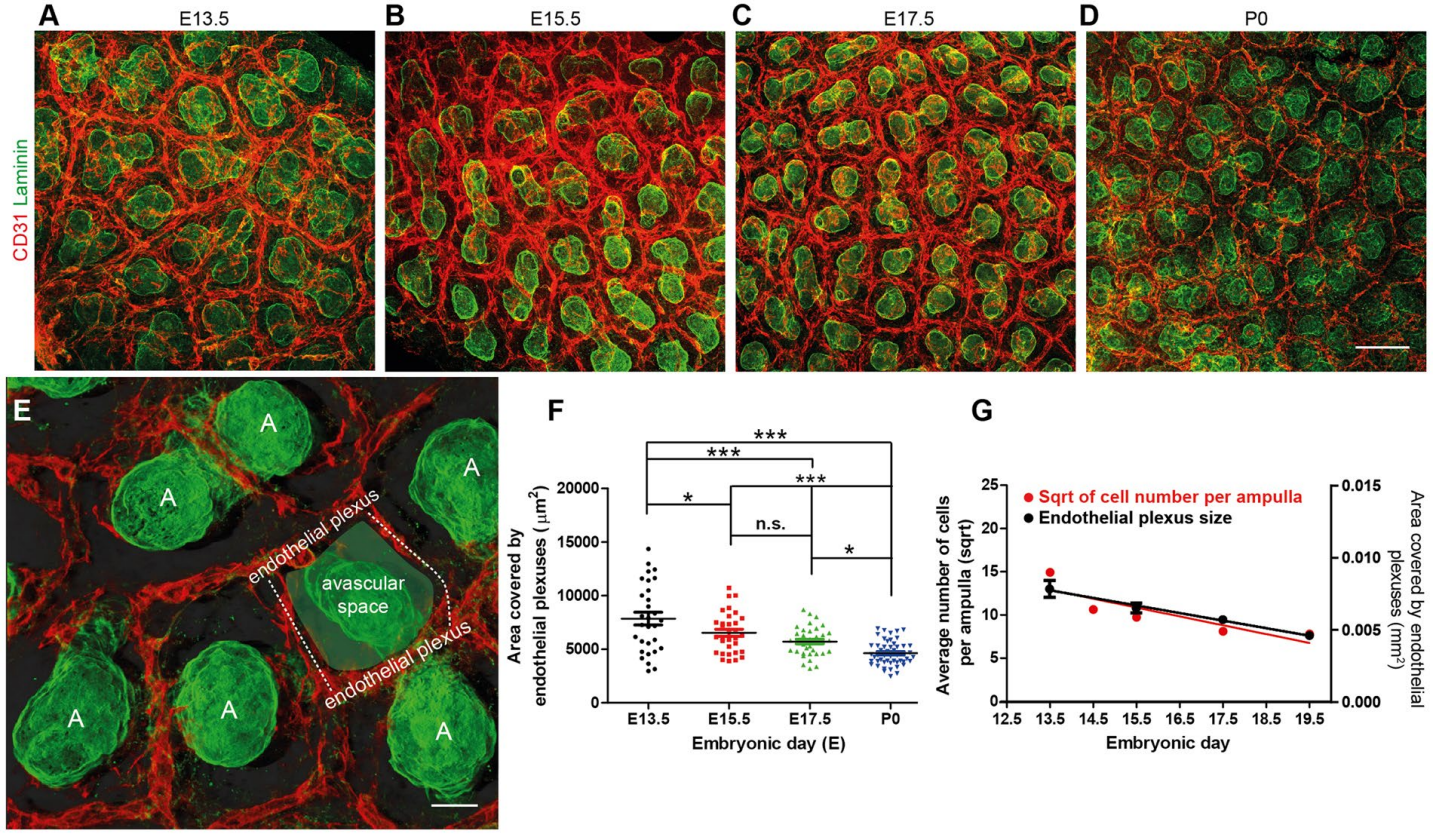
CD31^+^ plexuses form around ureteric bud ampullae. (**A–D**) Plexuses form around ureteric bud ampullae from E13.5–P0. (**E**) Image illustrating the organisation of endothelial plexuses around ureteric bud tips at E17.5 (blend projection prepared using IMARIS; A = ampulla). (**F**) Decreasing size of endothelial plexuses with embryonic age. (**G**) Slopes showing the similarity in the rate of decrease of endothelial plexus area and the square root of cell numbers per ampulla across embryogenesis. Scale bars: (A–D) = 100 µm; (E) = 20 µm.

By E12.5, the metanephric mesenchyme is vascularised (Fig. 2M). At this point in development, a region termed the nephrogenic zone forms around the periphery of the kidney. Within this zone, ureteric bud tips undergo rounds of divisions with concomitant nephron induction and formation^9^. From E13.5 and throughout embryonic development, polygonal endothelial networks, or endothelial plexuses, surrounded ureteric bud ampullae in the nephrogenic zone (Fig. 3A–E). By performing area coverage quantifications and subsequent statistical testing, we determined that there was a decrease in the average size of the endothelial plexuses with embryonic age (P < 0.001; Fig. 3F). Using linear regression analyses, a slope value for the ‘best-fit’ line was calculated that indicated that the average endothelial plexus area decreased by 0.0011 mm^2^ every two days from E13.5 to P0 (slope = −0.0011 mm^2^; 95% confidence interval = −0.0013 to −0.00076 mm^2^; r^2^ = 0.99). Additionally, there was a near perfect inverse-correlation between the average endothelial plexus size and embryonic age (r = −0.996; P < 0.01). We then examined the relationship between the rate of decrease of endothelial plexus areas and ampulla cell numbers (using data from ref. 33) across kidney development. Using a linear regression analysis, we did not find evidence that the slopes differ (*p* = 0.266; Fig. 3G).

Collectively, these results show that endothelial plexuses develop around ureteric bud tips throughout kidney development and that the area covered by endothelial plexuses decreases in a manner that corresponds to the decreasing cell numbers of ureteric bud ampullae during kidney development.

### Endothelial plexuses spatially arrange around the cap mesenchyme

**Figure 4:**
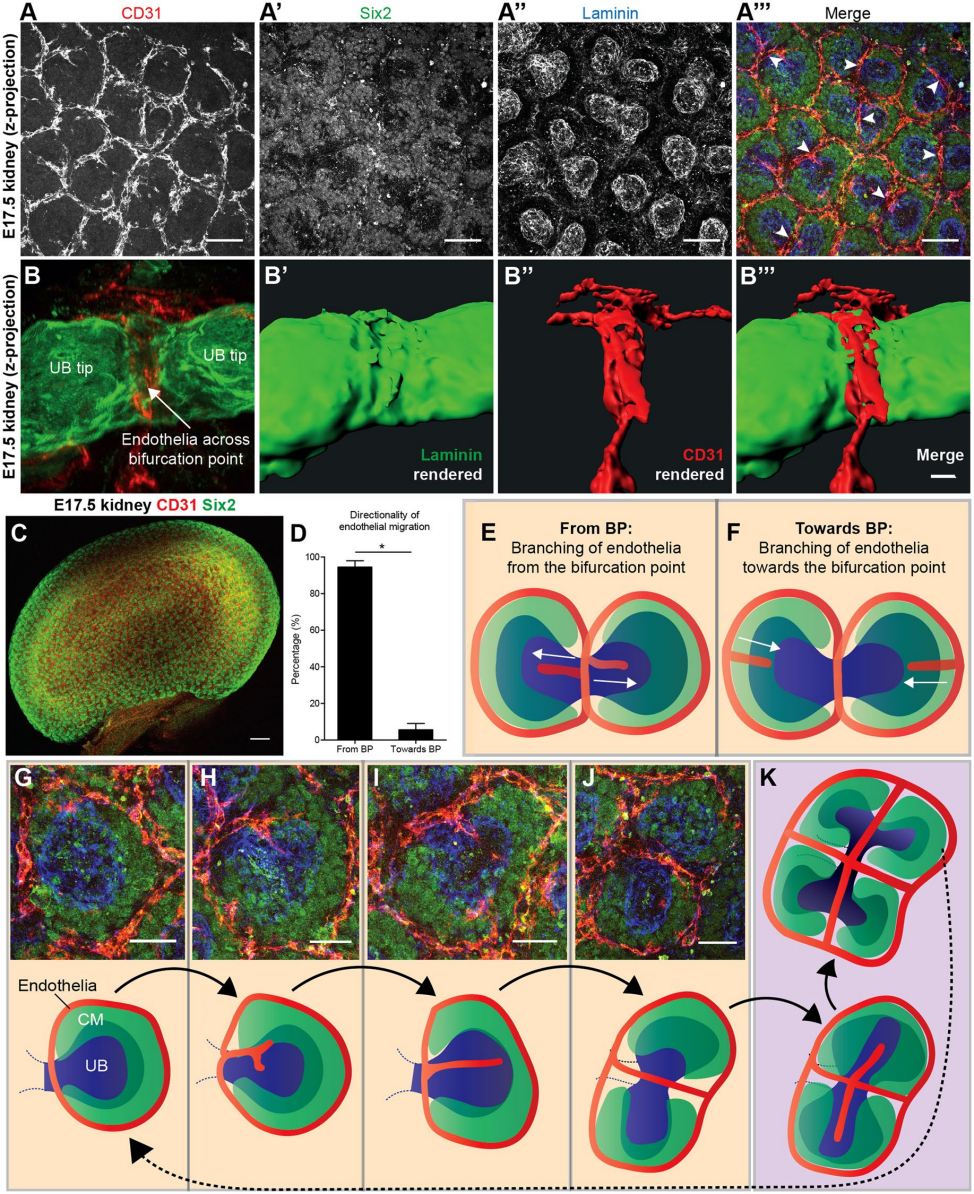
Endothelial plexus patterning and cap mesenchymal dynamics. (**A**-**A”’**) Endothelia arrange around the border of the cap mesenchymal populations and form across the bifurcation site of the ureteric bud (arrowheads). (**B**-**B”’**) Endothelial vessels develop in contact with the basement membrane at the ureteric bud bifurcation site. **B’**-**B”’** show 3-D rendered images produced from B. The CD31^+^ vessel crosses, and appears to distort, the basement membrane of the ureteric bud. (**C**) E17.5 kidney stained for CD31 and Six2. (**D**) Endothelia primarily send projections away from the bifurcation site (*p* = 0.0112; two-tailed Mann-Whitney test; *n* = 5). (**E**,**F**) Cartoon illustrating what is meant by endothelia branching *from* versus *towards* the bifurcation site. (**G**,**K**) Representative images and cartoons illustrating our cyclical model of endothelial patterning that occurs each generation of ureteric bud branching and cap mesenchymal splitting. CM, cap mesenchyme; UB, ureteric bud; BP, bifurcation point. Scale bars: A-A”’ = 50 µm; B-B”’, (G–K) = 20 µm; C = 200 µm.

Newly formed ureteric bud ampulla are coated by a population of cap mesenchymal cells^7^. As an ampulla matures, it bifurcates, leading to the generation of two new ampullae. This division is matched by the splitting of the overlaying cap mesenchymal population so that each newly formed ampulla inherits a population of cap mesenchymal cells. Our confocal images demonstrate that, as the cap mesenchymal population splits, CD31^+^ endothelia form between the two daughter cap mesenchymal populations at the bifurcation site (Fig. 4A-A”’). Endothelia also neatly arranged around the outer boundaries of cap mesenchymal populations (Fig. 4A-A”’). By analysing individual z-plane images we noted that the CD31^+^ cells at the bifurcation site were in extremely close proximity to the ureteric bud. 3D rendering of confocal z-projections of the E17.5 kidney consistently indicated that the crossing vessel contacted the surface at the bifurcation site and appeared to distort the ureteric bud basement membrane along its course (Fig. 4B-B”’).

To examine how new plexuses form as the kidney expands, we counted the amount of endothelial projections that extended *from* the bifurcation point in comparison to the amount of endothelial projections that formed *towards* the bifurcation point in E17.5 kidneys. We found that 94.44% (s.e.m. = ±3.52%; *n* = 5 kidneys) of endothelial projections extended away *from* the bifurcation site (Fig. 4D–F). These projections extended before conjoining with other endothelia to form new vascular plexuses when the cap mesenchymal populations split (Fig. 4G–K). These findings demonstrate the timing of blood vessel development across the bifurcation site: that is, when the cap mesenchymal cells vacate this area, endothelia migrate across it. Endothelia project in the same orientation at E14.5 (Supplementary Fig. 4A–C), supporting the concept that this process is cyclical, reoccurring with each new generation of ureteric bud branching. Together, these data illustrate that the patterning of endothelia within the nephrogenic zone relates to the arrangement of cap mesenchymal cells.

### Endothelial plexuses form around the ureteric bud and cap mesenchyme *in vitro*

**Figure 5:**
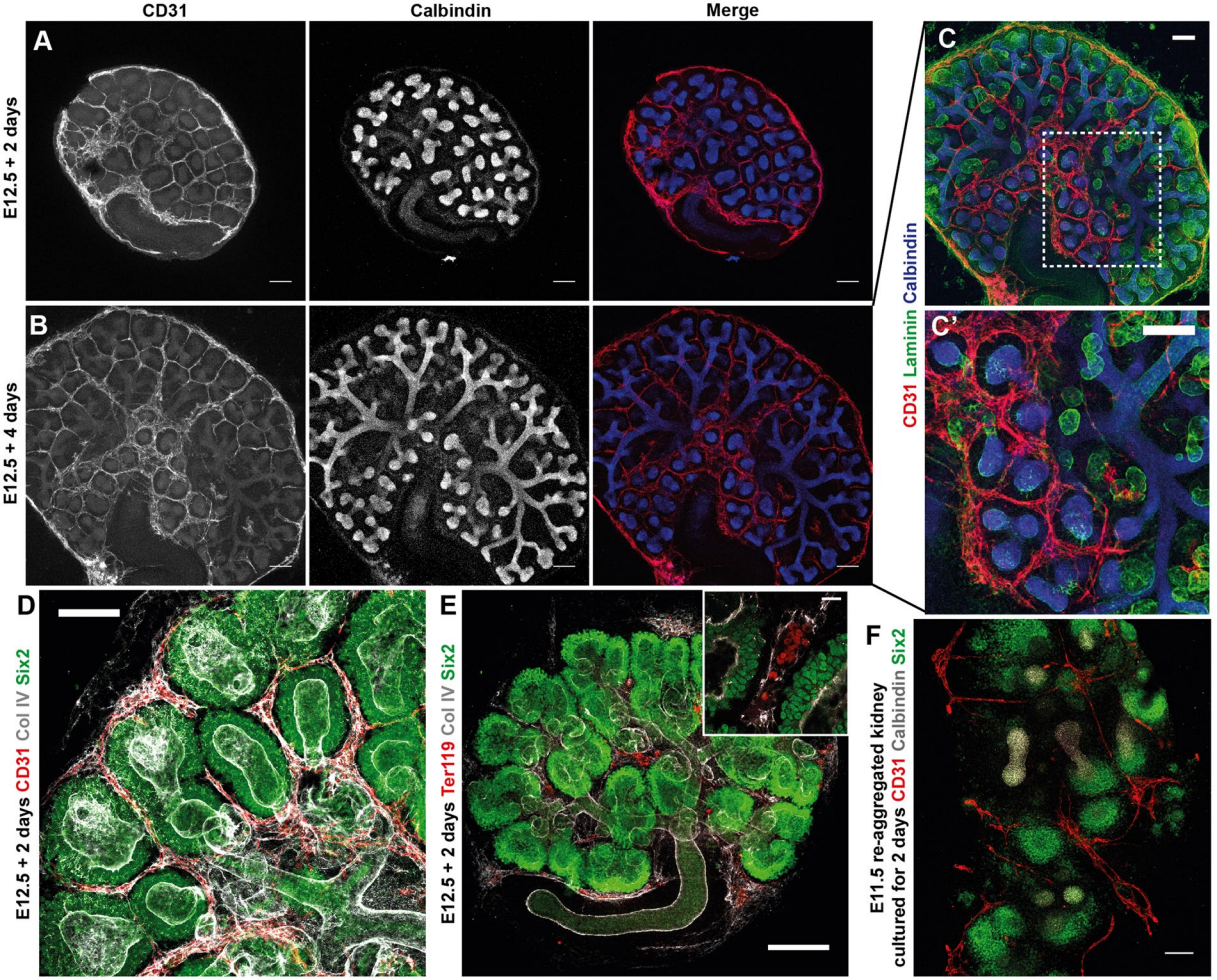
Endothelial plexuses form around ureteric bud tips and the cap mesenchyme *in vitro*. (**A**) Representative images of an E12.5 + 2 days and (**B**) + 4 days cultured kidney. (**C**-**C’**) 4 days cultured kidney showing that plexuses formed in regions where ureteric bud tips are present, but not in regions devoid of ureteric bud tips. Note in C’ that endothelial plexuses do not form around the epithelia of the developing nephrons (green), they only form around ureteric bud epithelia (blue). (**D**) Endothelial plexuses formed around cap mesenchymal populations in culture. (**E**) Ter119^+^ erythroid cells were present in blood vessels in cultured kidneys. (**F**) Endothelia formed around cap mesenchymal populations in re-aggregated kidneys. Scale bars: 100 µm.

We investigated endothelial patterning in cultured E12.5 kidneys and re-aggregated kidneys to determine whether the patterning ‘rules’ we have shown *in vivo* are also obeyed *in vitro*. By culturing E12.5 kidneys for 2 and 4 days, we demonstrated that endothelial plexuses formed around the ureteric bud tips and cap mesenchymal populations in culture (Fig. 5A–D). Endothelia specifically formed plexuses around the epithelia of the ureteric bud, but not the epithelia of nephrons (Fig. 5C-C’). Additionally, Ter119^+^ erythroid cells were found within basement membrane enclosed vessels in the cultured kidneys, indicating that the vessels in culture were capable of carrying blood (Fig. 5E).

We then investigated if plexuses would form around ureteric bud tips and cap mesenchymal niches in kidneys re-aggregated from suspensions of renogenic stem cells (as produced in ref. 34), and determined that endothelia also pattern around ureteric bud tips and cap mesenchymal populations in this model (Fig. 5F).

These data show that endothelial plexuses formed around cap mesenchymal and ureteric bud cells in various *in vitro* models.

### The plexus endothelia are vascular, erythrocyte carrying, enclosed by a basement membrane, and can be traced back to the renal artery

**Figure 6:**
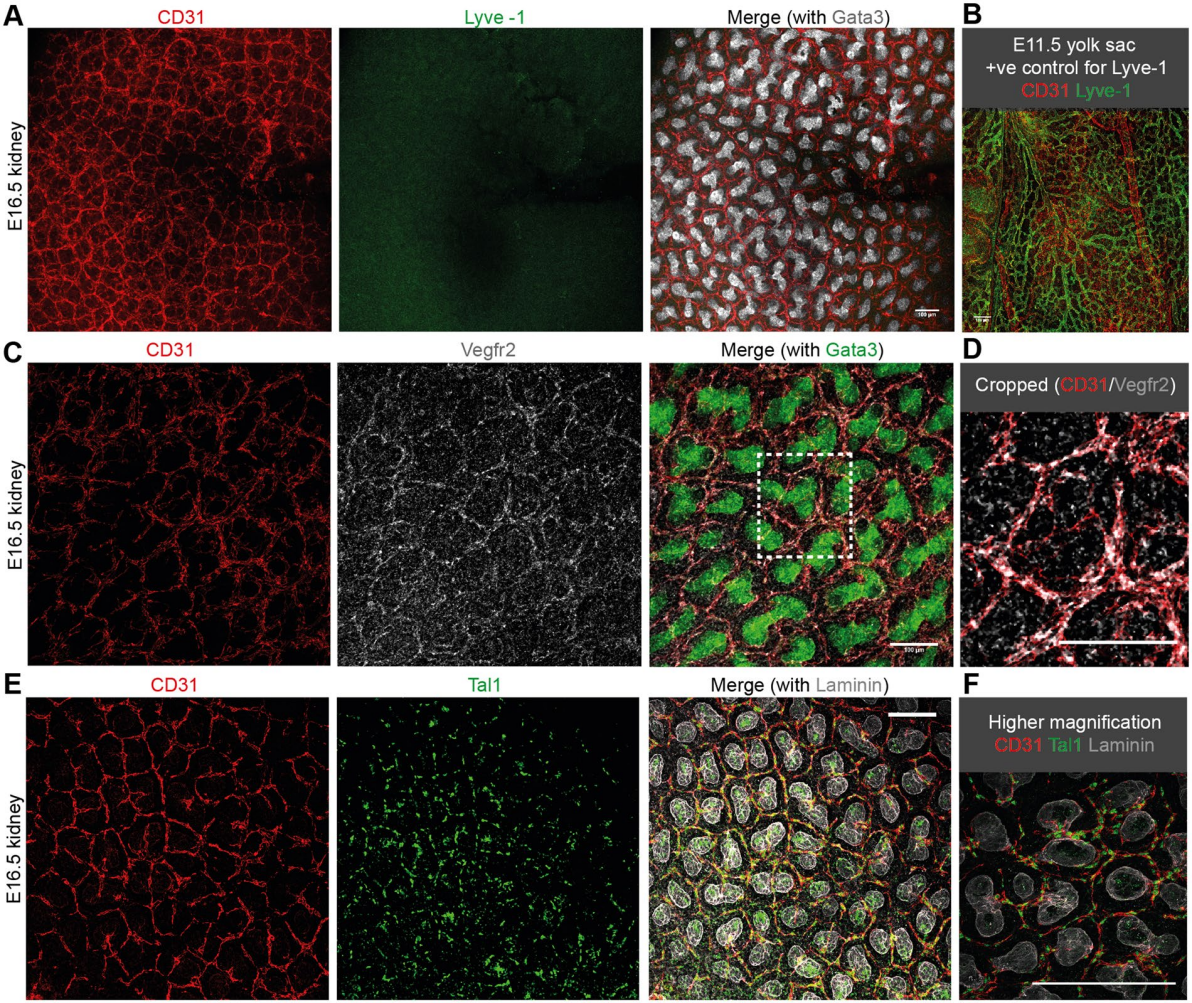
Characterisation of plexus endothelia. (**A**,**B**) Plexus endothelia are Lyve-1^−^. (**C**,**D**) Plexus endothelia are Vegfr2^+^. (**E**,**F**) Plexus endothelia are SCL/Tal1^+^. Due to high antibody background, the Despeckle tool in ImageJ was used to eliminate background signal in the Vegfr2 channel in C-D and the SCL/Tal1 channel in E-F. Scale bars: 100 µm.

Since CD31 is a pan-endothelial marker^35^, it does not discriminate between lymphatic or vascular endothelia. To examine if the endothelia are vascular, we performed co-localisation analyses of CD31^+^ cells with a specific marker of lymphatic endothelia (Lyve-1) and a typically vascular endothelial marker (Vegfr2). The plexus endothelia were Vegfr2^+^ (Fig. 6A,B), but Lyve-1^−^ (Fig. 6C; E11.5 yolk sacs were stained alongside kidneys as a positive control for anti-Lyve-1, Fig. 6D).

Previous work has shown that SCL/Tal1^+^ cells exist within the kidney and act as progenitors of blood cells and endothelia via haemovasculogenesis^24^. We examined whether any SCL/Tal1^+^ cells were present within the nephrogenic zone that might give rise to renal endothelia. We identified many SCL/Tal1^+^ nuclei within the nephrogenic zone. The nuclear SCL/Tal1^+^ staining always coincided with the fully differentiated CD31^+^ plexus endothelia, but we did not observe any SCL/Tal1^+^ cells that were not also CD31^+^ (Fig. 6E,F). Collectively, we determined that the plexus endothelia are CD31^+^Vegfr2^+^Lyve-1^−^SCL/Tal1^+^, supporting the hypothesis that the plexus endothelia are vascular.

**Figure 7:**
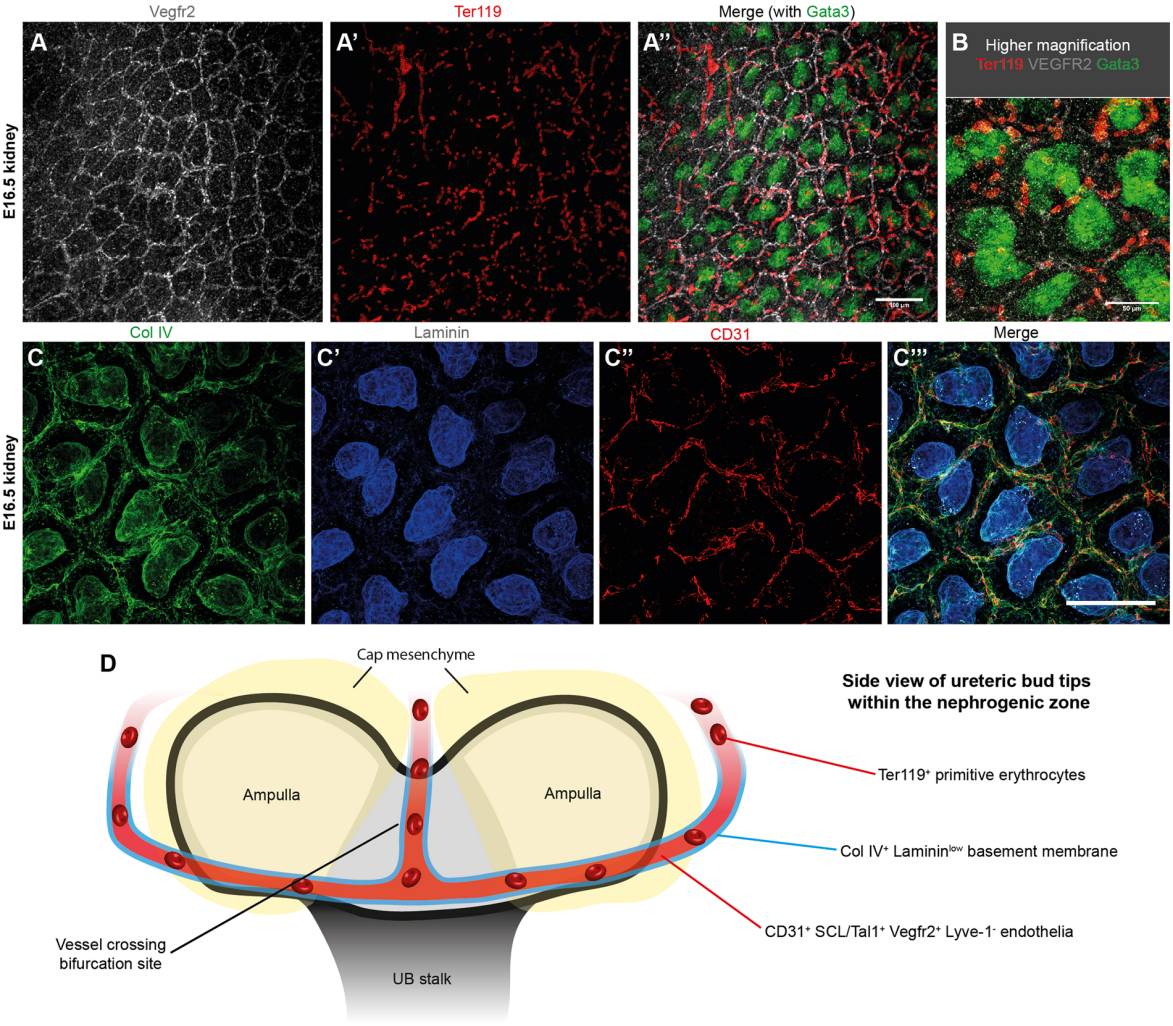
The vascular plexuses contain erythrocytes and are enclosed by a collagen IV^+^ and laminin^low^ basement membrane. (**A**,**B**) Plexus endothelia carry Ter119^+^ erythrocytes. Due to high antibody background, the Despeckle tool in ImageJ was used to eliminate background signal in the Vegfr2 channel. (**C**-**C”’**) The vascular plexuses are collagen IV^+^ and laminin^low^ in E16.5 kidneys. (**D**) Cartoon depicting the side-view of the ureteric bud surrounded by cap mesenchymal populations and vascular plexuses. Scale bars: 100 µm (except for B, where it represents 50 µm).

To provide further evidence that the endothelia are vascular, we examined whether erythroid cells were being carried in the vessels. The plexus vessels contained Ter119^+^ erythroid cells at E14.5 and E16.5 (Fig. 7A,B; Supplementary Fig. 4D). This was surprising as previous work had suggested that these peripheral vessels are of a vasculogenic origin, are predominantly non-perfused^17^, and do not have a lumen at this stage (in rabbit ref. 36). Therefore, we expected an absence of erythroid cells. Unlike the erythroid cells at E11.5 and E12.5, most erythroid cells at E16.5 were enucleated (94% were enucleated [*n* = 100 cells]; the 95% confidence interval is ±4.39%) which agrees with previous work showing that most erythroid cells have matured into primitive erythrocytes by this stage of mouse embryogenesis^31, 32^.

We lastly examined if the vascular plexuses were enclosed by a basement membrane. We found that a collagen IV basement membrane surrounded the vascular plexuses (Fig. 7C-C”’) and observed low levels of staining for the basement membrane marker laminin (Fig. 7C’; Supplementary Fig. 5; Supplementary Fig. 6).

Notably, at the various ages tested, all CD31^+^ endothelial cells could be traced to other CD31^+^ endothelial vessels within the kidney, all the way back to the renal arteries (*n* = 50 traced vessels from E13.5–17.5; Movie 7). Even the most peripheral endothelia were connected to the pre-existing vasculature. Together, these results demonstrate that the vascular plexuses contain erythrocytes, are enclosed by a basement membrane, and are connected to the pre-existing blood vessels.

## Discussion

In this study, we took high-resolution images of whole-mount normal kidneys that had developed *in vivo*. By doing so, we provide a spatiotemporal analysis of endothelial development in the CD-1 mouse kidney from the initiation of its development to birth. This has improved our understanding of the first steps of kidney vascularisation, and has provided a conceptual model for endothelial patterning within the nephrogenic zone of the kidney.

The cyclical patterning of endothelial plexuses that we have shown during development (see Fig. 4G–K) will likely facilitate the proper assembly of the adult kidney’s vasculature. Future studies should investigate the consequences of disrupting this patterning on adult kidney function. This patterning may also serve important roles in the development of other renal structures, such as nephrons. Oxygen promotes nephrogenesis^17, 37^, and the erythrocytes carried within the vascular plexuses may provide oxygen to drive nephron formation.

Our model of vascular plexus formation from pre-existing blood vessels is based on several findings. First, at no age did we observe any endothelia that were not already in connection with the pre-existing vasculature. Indeed, we show that even the most peripheral CD31^+^ endothelia can be traced all the way back to the renal artery. Second, SCL/Tal1^+^ endothelia were always also CD31^+^. Should vasculogenesis occur at the periphery of the kidney, we would expect to observe isolated SCL/Tal1^+^CD31^−^ angioblasts within this region^38^. Third, the fact that the peripheral vascular plexuses were enclosed by a basement membrane and carried erythrocytes is more consistent with angiogenesis than with vasculogenesis. However, the erythrocytes within the plexuses could arise in part, or entirely, via haemovasculogenesis^24, 39^, rather than being transported there via the circulation.

A major finding of this study is that endothelia do not enter the Six2^+^ cap mesenchyme, and instead arrange around it. Even prior to E11.5, when the entirety of the metanephric mesenchyme is Six2^+^, endothelia arrange around, rather than through this population of cells (possibly explaining why the metanephric mesenchyme remains avascular at its earliest stages, despite it being surrounded by blood vessels). This may be a result of the cap mesenchyme releasing an anti-angiogenic signal, or signals, that inhibit endothelial migration into the cap mesenchyme. Exposing the underlying mechanisms of endothelial patterning around the cap mesenchyme will greatly enhance our understanding of how the complex architecture of the renal vasculature develops.

Another intriguing result is that endothelial vessels form across, and it contact with, the bifurcation site of the ureteric bud (when the ureteric bud branches, and the cap mesenchymal populations split). In many branching organs, cell types that cross the bifurcation site play important roles in controlling branching stereotypy through a mechanical or signalling role^40–42^. It is possible that the crossing endothelia may play a similar role in ureteric bud branching in the kidney.

A long-standing question concerning kidney vascularisation is, whether the blood vessels form through angiogenesis, vasculogenesis, or a combination of both. In culture, the potential for extrinsic blood vessels to invade the kidney is eliminated, and factors that drive angiogenesis, such as blood flow^43^, can no longer influence endothelial organisation (perhaps explaining why glomeruli are predominantly avascular in culture)^44, 45^. Thus, kidney culture conditions might not offer an appropriate environmental milieu to study blood vessel formation, as it would seemingly thwart angiogenesis. However, we and others have shown that endothelia from the early embryonic kidney can survive in culture^25, 46^, and live tracing experiments of cultured kidneys from endothelial cell-specific *Tie1Cre*;*R26R* ^*YFP*^ reporter mice illustrated that vessels develop from the pre-existing endothelia through a mechanism that is consistent with sprouting angiogenesis^25^.

In support of renal vascularisation by angiogenesis, cross-transplantation experiments have consistently indicated that the kidney can attract the invasion of extrinsic blood vessels^47–49^. However, these studies did not examine whether endogenous vessels were also developing *de novo*, so were weighed against revealing vasculogenesis.

Other cross-transplantation studies have focused on investigating the kidneys ability to form blood vessels intrinsically by probing for specific genetic markers of endogenous endothelia^24, 28, 47^. In these studies, donor vessels developed, and often these vessels were assumed to form via vasculogenesis, as the transplanted donor kidneys were believed to be avascular at the time of grafting (for example, E12 kidneys^26, 28, 47, 50^ and E12.5 kidneys^24^). However, based on our results, the transplanted kidneys would already have formed endothelial vessels prior to transplantation. In reality, it would be extremely difficult to transplant an embryonic kidney at any age (with the metanephric mesenchyme and ureteric bud) without also transplanting contaminating donor endothelia, as noted by Loughna *et al*.^21^. As the endothelia in the kidney are clonal^51^, it is possible that the pre-existing donor endothelia could have survived and contributed to blood vessel formation via angiogenic processes, rather than via vasculogenesis. This might explain why chimeric endothelial vessels, with host and donor endothelia, are often observed in cross-transplanted kidneys^14, 26, 28, 52^.

Regardless of the process of blood vessel formation, there is convincing evidence that endogenous endothelial cells do exist within the kidney^22, 24, 25^, and our results do not eliminate the possibility that vasculogenesis may contribute to the renal vasculature in some as yet undetermined way.

An example of a renal cell type with the capacity to differentiate into endogenous endothelia are the Foxd1^+^ stromal cells^22, 25^. Within the nephrogenic zone, vascular plexus formation occurs within the Foxd1^+^ populations of cells^22^. Perhaps the Foxd1^+^ cells that become endothelial are induced to differentiate and are recruited into the developing vascular plexuses via a process that combines angiogenic (vessels forming from pre-existing vessels) and vasculogenic principles (differentiation, migration, and coalescence of endogenous endothelia).

Overall, we show that the first blood vessels enter the kidney from capillaries situated within a peri-Wolffian mesenchymal region. We suggest that these vessels migrate to the periphery of the kidney by ~E12 and start organising as vascular plexuses around the ureteric bud/cap mesenchyme as the nephrogenic zone forms. As rounds of ureteric bud branching take place, new plexuses form cyclically throughout the remainder of embryogenesis. Future studies should examine the exact molecular mechanisms of vascular plexus formation and investigate how the plexuses are remodelled as the kidney develops.

## Methods

### Animals

Embryonic tissues were obtained from healthy CD-1 mice that were killed by qualified staff of the UK Home Office-licenced animal house following the guidelines set under Schedule 1 of the UK Animals (Scientific Procedures) Act 1986. All experiments were approved by the University of Edinburgh and performed in accordance with the institutional guidelines and regulations.

### Dissection and organ culture

Embryonic kidneys were dissected using methods previously described^53^. For studies of normal development, kidneys were processed for immunofluorescence directly after dissection. For culture experiments, dissected kidneys were placed in Saxén-style culture^54^ for up to 4 days. Briefly, kidneys were placed on polycarbonate membrane filters (5 µm pores; Sigma, P9699-100EA), with these filters being supported by a metal grid in 35 × 10 mm culture plates (Greiner, 627–160). Kidneys were cultured in 2–2.5 ml of kidney culture medium (Minimum Essential Medium Eagle [Sigma, M5650] supplemented with 1% penicillin/streptomycin [Sigma, P4333] and 10% foetal calf serum [Invitrogen, 10108165]). Kidneys were grown at 37 °C in a 5% CO_2_ environment. Medium was changed every 48 hours.

### Generation of re-aggregated kidneys

Re-aggregated kidneys were generated as previously described^34^. Briefly, eight E11.5 kidneys were dissected and pooled. The pooled kidneys were trypsinised for 1 min at 37 °C in a 5% CO_2_ environment. Cells were manually dissociated and passed through a 40 µm pore sized cell strainer (Falcon, 352340). The dissociated cells were pelleted by centrifugation for 1 min 30 secs at 3000 RPM, and placed in Saxén-style culture for the indicated times. The ROCK inhibitor, glycyl-H1152-dihydrochloride (1.25 µM), was added to the medium for the initial 24 hours of culture.

### Conventional whole-mount immunofluorescence

Kidneys were fixed in methanol (pre-cooled at −20 °C) for 1 hr. Kidneys were then rinsed in 1x PBS (3 × 30 mins), and blocked with 1x PBS with 5% BSA (Sigma, A9647) and 10% donkey serum (Sigma, D9663) for 1 hr-overnight. Kidneys were incubated with primary antibodies overnight at 4 °C (for antibody information see Supplementary Table 1). Kidneys were then rinsed in 1x PBS (3 × 1 hr) and subsequently incubated in secondary antibodies for 2–4 hrs at room temperature or overnight at 4 °C. Finally, kidneys were washed in 1x PBS (4 × 1 hr), before being mounted onto glass slides using Vectashield (Vectorlabs, H1000) as a mounting medium.

### BABB clearing whole-mount immunofluorescence

Samples were fixed in Dent’s bleach for 2 hrs, then stored in Dent’s fixative at −20 °C. Samples were washed with 1x PBS-T (3 × 30 mins) and blocked with 1x PBS-T with 5% BSA, 10% donkey serum, and 5% DMSO (AppliChem, A3672-0100) overnight. Samples were incubated with primary antibodies diluted in the blocking buffer for 1–5 days at 4 °C. Samples were then washed in 1x PBS-T for 3 × 2 hrs. Samples were subsequently incubated in secondary antibodies for 24 hrs at 4 °C diluted in the blocking buffer, were washed for 4 × 1 hrs with 1x PBS-T, and dehydrated in a series of 15 min methanol dehydration steps (20%, 50%, 75%, and 100%). They were then cleared in a glass vial in 50% BABB (1:2 benzyl alcohol/benzyl benzoate)/Methanol and then 100% BABB until transparent. Finally, samples were mounted onto a slide and covered with a drop of BABB prior to imaging.

### Imaging

Images were generated using the Nikon A1R confocal microscope with NIS elements software. Objectives of 4–60x were used. The objective lens was oil-immersed from 40x upwards. ImageJ (FIJI) and IMARIS were used to process and analyse images.

### Vascular plexus area measurements

The area covered by each vascular plexus was defined using the freehand selections tool in ImageJ. Using this tool, each region was traced three times and the mean of the three measurements (determined using Analyse > Measure) was taken as the area covered by that vascular plexus. Subsequently, the area of each vascular plexus at each embryonic age was pooled so that a mean could be calculated and compared for each age. To compare the decrease in plexus area to the decrease in ampulla size, the square root of the number of cells per 3-D ampulla (using data from ref. 33) was related to the area calculation.

### Statistical analyses and data presentation

For the vascular plexus area studies, D’Agostino-Pearson omnibus normality tests were used followed by a one-way ANOVA with the post-hoc Tukey’s Multiple Comparison Test. For the directionality of endothelial migration study, a two-tailed Mann-Whitney test was employed. 95% confidence intervals for percentage counts were calculated using the formula t = 1.64SQRT(pq/n) + 1/2n, where t is the 95% confidence interval, p is the proportion positive, q is 1−p, and n is the number examined. GraphPad prism (version 5) was used for data analyses and graph preparation. Adobe illustrator CC 2015 was used to prepare figures. IMARIS (version 8.2.1) and Adobe premiere pro CC 2015 were used to prepare videos.

### Data availability

The datasets generated and analysed during the current study are available from the corresponding author on reasonable request.

## Electronic supplementary material

Supplementary Information

Movie 1

Movie 2

Movie 3

Movie 4

Movie 5

Movie 6

Movie 7
