# Supplementary material for: Cycles of vascular plexus formation within the nephrogenic zone of the developing mouse kidney
Source: Sci Rep. 2017 Jun 12;7:3273. doi: 10.1038/s41598-017-03808-4 (PMC5468301; doi:10.1038/s41598-017-03808-4)
Supplement: Supplementary file 1 — Supplementary Information [file 41598_2017_3808_MOESM1_ESM.pdf]

Supplementary text and data for:

Cycles of vascular plexus formation within the nephrogenic zone of the developing mouse kidney

Authors: David A. D. Munro[1*], Peter Hohenstein[2], and Jamie A. Davies[1]

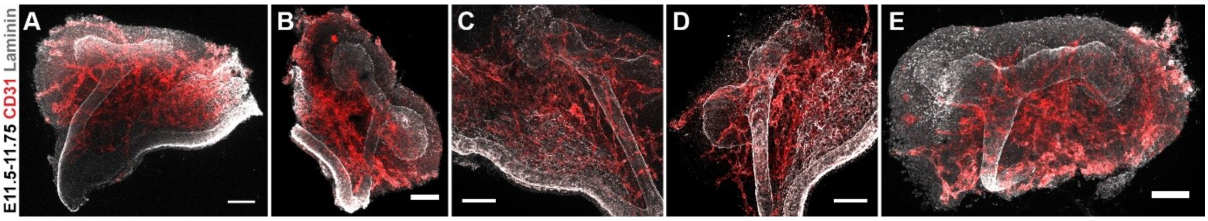

Supplementary Figure 1. Considerable vascularisation of the kidney occurs between E11.5 and E11.75 (as the cross-stroke of the ‘T’ elongates). Scale bars: 100 μm.

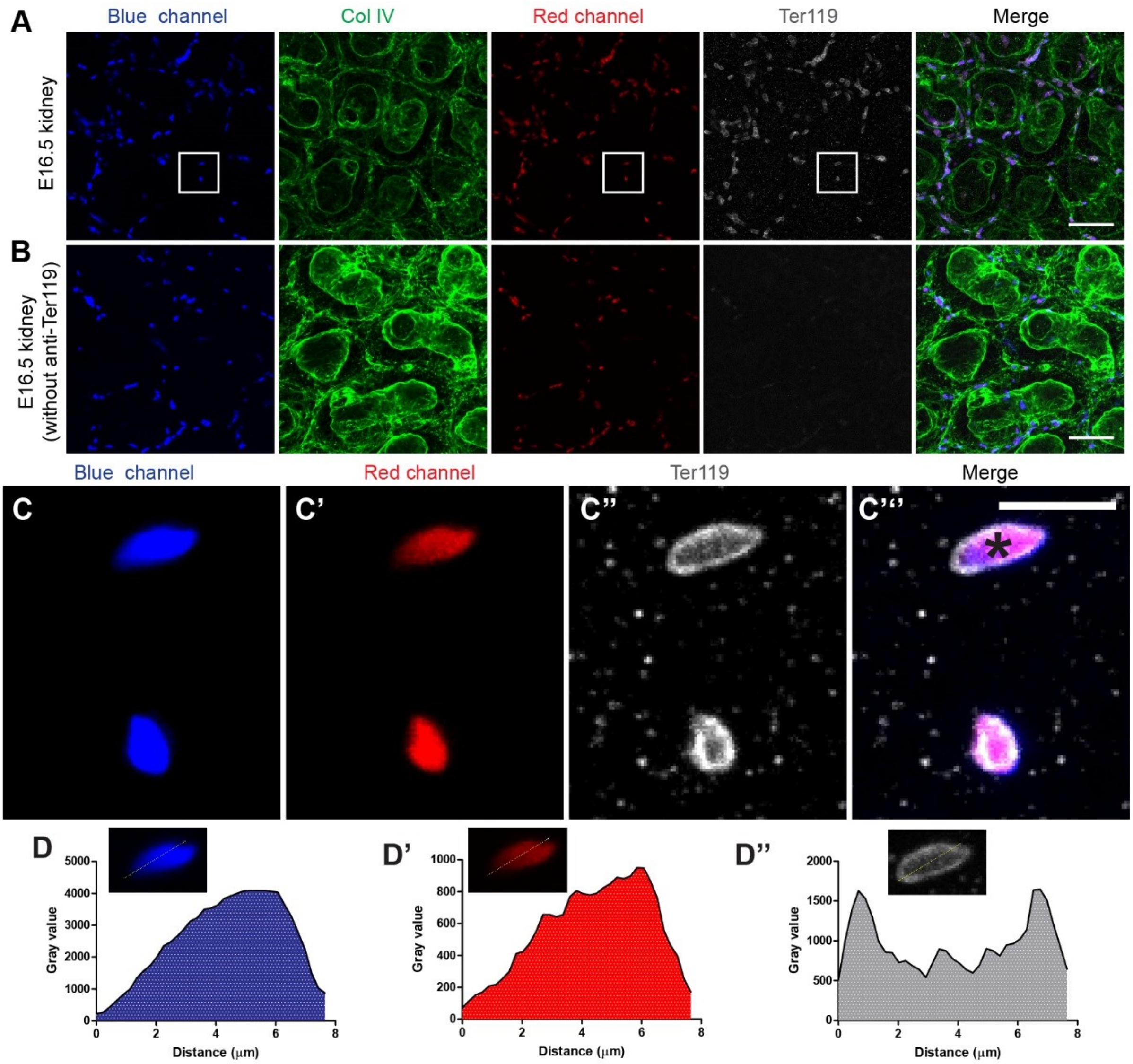

Supplementary Figure 2. Autofluorescence by erythroid cells in bleached tissue. (A) In bleached E16.5 kidneys, cells autofluoresce under visible light and co-localise with the erythroid cell marker, Ter119. No antibodies were used to produce fluorescence in the blue or red channels. **(B)** Shows the same as A, but without anti-Ter119. Images from A and B were taken using identical microscope settings. **(C-C''')** Cropped image of two cells that are highlighted by the white box in A. Note the different fluorescence profile of the cells in the far-red channel (Ter119) compared to the blue and red channels. **(D-D'')** Gray value profiles of the cell in C''' that is marked by the asterisk (*****; data plotted using Analyse>Plot Profile in ImageJ). In D and D', the gray value profiles are alike (the result of intracellular heme acting as a chromophore). In D'', the gray value profile has two peaks, representing fluorescence at each side of the plasma membrane (Ter119 binds to a molecule that associates with glycophorin A on the erythroid cell membrane). Scale bars: A-B = 100 µm; C-C''' = 10 µm.

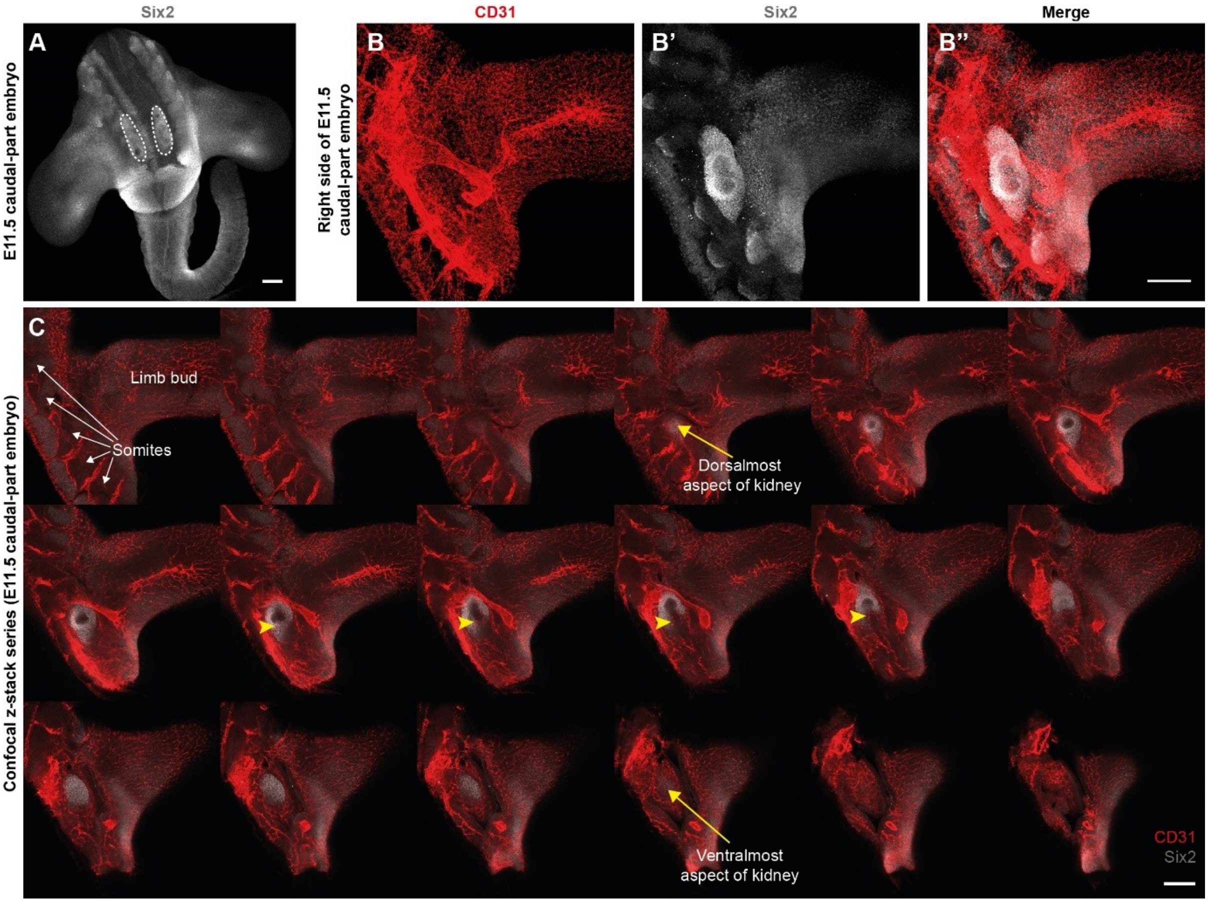

Supplementary Figure 3. Location of the kidneys in the E11.5 mouse embryo. (A) The E11.5 kidneys sit in the caudal-part of the mouse embryo at the level of the hind limb buds. **(B-C)** The kidney is positioned in a densely vascularised region of the caudal-part mouse embryo and is surrounding by small capillaries and major arteries. Yellow arrowheads show the vascular ring and peri-ureteral blood vessels (note that these vessels connect to the adjacent major arteries). Scale bars = 200 µm.

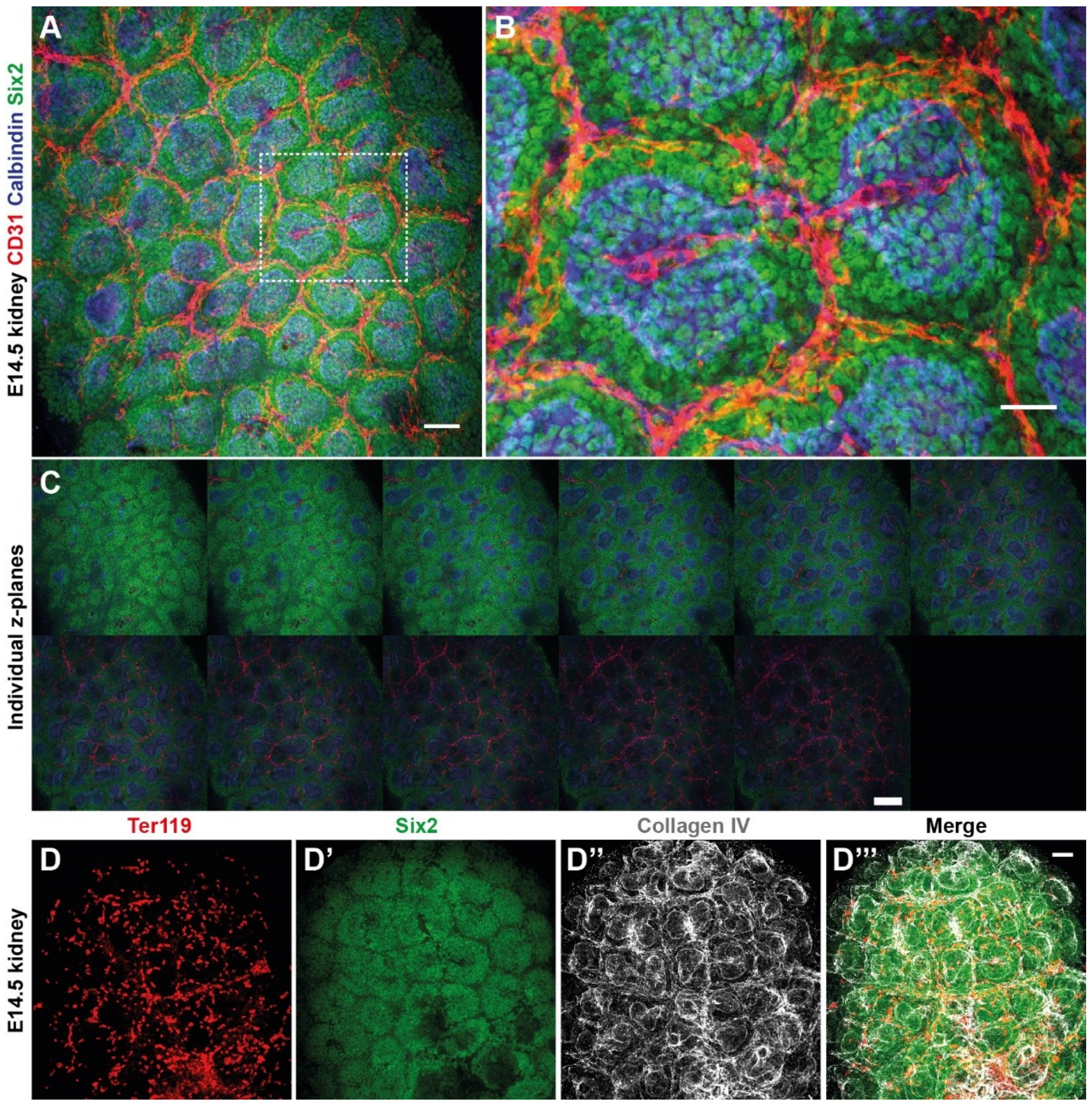

Supplementary Figure 4. Vascular plexuses and erythroid cells are present around cap mesenchymal populations at E14.5. (A-B) At E14.5, endothelia branch from the bifurcation site of the ureteric bud to form a new plexus. **(C)** Individual z-planes (from the z-stack in A-B) show that the green and red cells in A-B are not overlapping (the green cap mesenchymal cells sit above the blue ureteric bud tip cells). **(D-D’’’)** Erythroid cells are present within the peripheral vascular plexuses at E14.5. Scale bars: A and C = 100 µm; B = 20 µm; D-D’’’ = 50 µm.

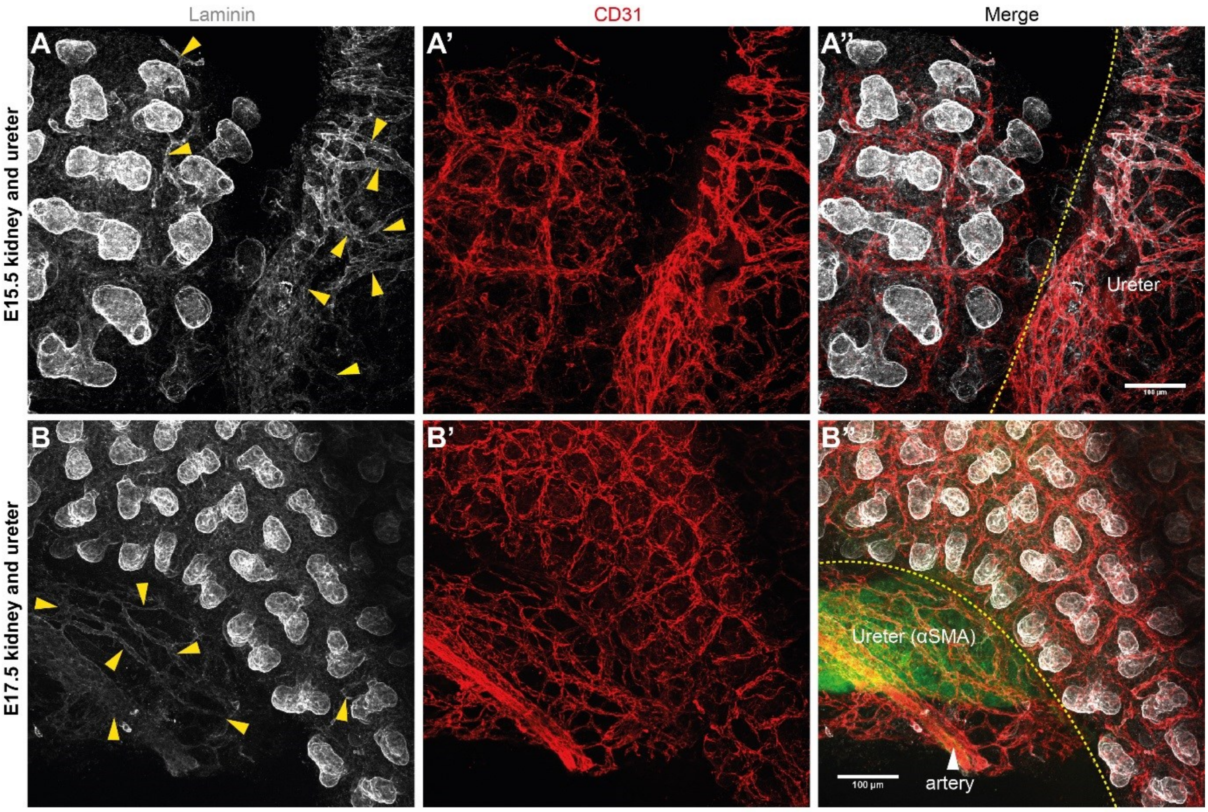

Supplementary Figure 5. The basement membrane of the vascular plexuses differs to that of other blood vessels in the kidney and ureter. (A-A'') At E15.5, the basement membrane of most vascular plexuses have low levels of laminin compared to ureteral blood vessels. **(B-B'')** Shows the same as A, but at E17.5. Yellow arrowheads show examples of laminin^{high} blood vessels. Scale bars = 100 μm.

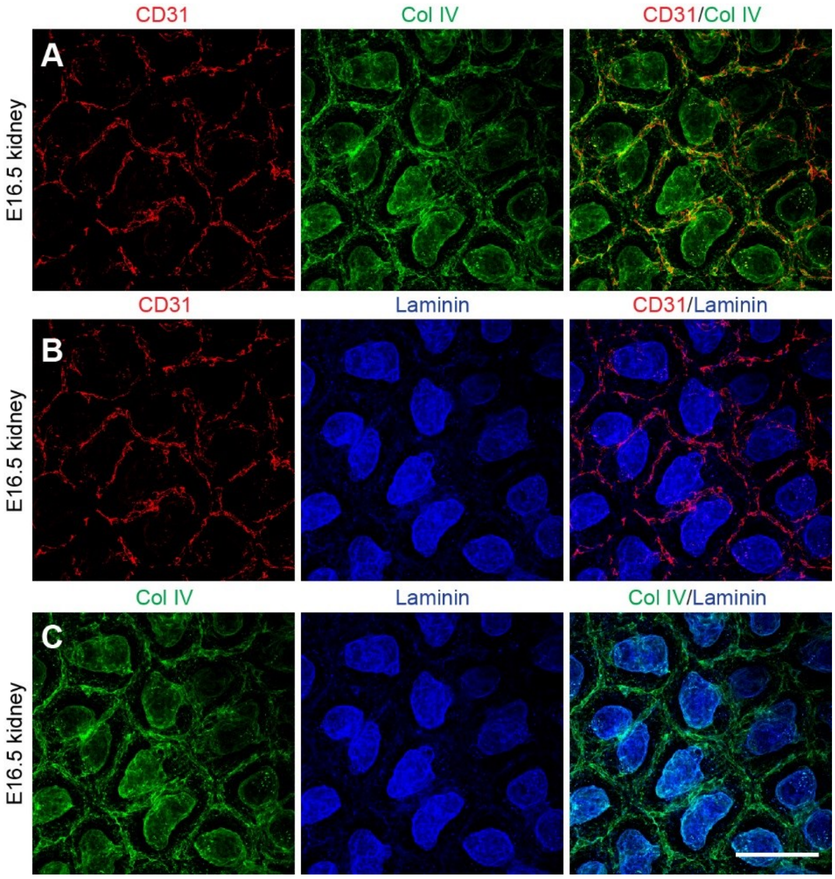

Supplementary Figure 6. The endothelial plexus basement membrane is collagen IV⁺ and laminin^low in E16.5 kidneys. **(A-C)** Show all combinations of CD31, Col IV, and laminin staining. Scale bar = 100 µm.

Supplementary Table 1. Antibodies used.

Primary antibodies	Working dilution	Clonality	Supplier (Cat. Number)
Rat anti-mouse CD31	1 in 100	Monoclonal	BD Pharmingen (550274)
Rabbit anti-mouse laminin	1 in 100	Polyclonal	Sigma (L9393)
Mouse anti-mouse calbindin	1 in 100	Monoclonal	Abcam (ab9481)
Mouse anti-mouse pan-cytokeratin	1 in 100	Monoclonal	Sigma (C2562)
Rabbit anti-mouse Six2	1 in 200	Polyclonal	Proteintech (11562-1-AP)
Rabbit ant-mouse VEGFR2	1 in 100	Monoclonal	Cell signalling technology (55B11)
Goat anti-human Gata3	1 in 200	Polyclonal	R&Dsystems (AF2605)
Rabbit anti-mouse Lyve-1	1 in 100	Polyclonal	Abcam (ab33682)
Rat anti-mouse Ly76 (TER-119)	1 in 100	Monoclonal	Abcam (ab91113)
Goat anti-human collagen IV	1 in 100	Polyclonal	Merckmillipore (AB769)
Goat anti-mouse SCL/Tal1	1 in 100	Polyclonal	Santa cruz (ab24870)

Conjugated primary antibodies	Working dilution	Clonality	Supplier (Cat. Number)
FITC-conjugated mouse anti-mouse actin, α-smooth muscle	1 in 100	Monoclonal	Sigma (F3777)

Conjugated secondary antibodies	Working dilution		Supplier (Cat. Number)
AlexaFluor 350 donkey anti-mouse	1 in 200		Life Technologies (A10035)
AlexaFluor 488 donkey anti-mouse	1 in 200		Life Technologies (A21202)
AlexaFluor 488 donkey anti-rabbit	1 in 200		Life Technologies (A21206)
AlexaFluor 647 donkey anti-rabbit	1 in 200		Life Technologies (A31573)
AlexaFluor 488 donkey anti-goat	1 in 200		Life Technologies (A11055)
AlexaFluor 647 donkey anti-goat	1 in 200		Life Technologies (A21447)
AlexaFluor 594 chicken anti-rat	1 in 200		Life Technologies (A21471)
AMCA Goat anti-rabbit	1 in 100		Abcam (ab123435)
AMCA horse anti-mouse	1 in 100		VECTOR (CI-2000)

Movie titles and captions

Movie 1. Dorsal view of the blood vessels in the E11.75 kidney. Note the vascular ring that had formed around the top of the ureteric bud stalk. Scale bar: 100 µm.

Movie 2. Position of the E11 kidney in the caudal portion of the mouse embryo. 3-D rendering was performed using IMARIS. Scale and labels are provided in the Movie.

Movie 3. Stack of z-plane images of the E11 kidney in the caudal portion of the mouse embyro. Scale bar: 100 µm.

Movie 4. Primitive erythroblasts in the E11.5 kidney. Scale and labels provided in the Movie.

Movie 5. Position of the E11.5 kidney in the caudal portion of the mouse embryo. 3-D rendering was performed using IMARIS. Scale and labels are provided in the Movie.

Movie 6. Stack of z-plane images of the E11.5 kidney in the caudal portion of the mouse embyro. Scale bar: 100 µm.

Movie 7. Tracing endothelia from the vascular plexuses to the renal arteries, and tracing from renal arteries to plexus endothelia, in the E17.5 kidney. The coloured stars represent different examples of vessels that were traced.
